# A Novel De Novo *SP6* Mutation Causes Severe Hypoplastic Amelogenesis Imperfecta

**DOI:** 10.3390/genes12030346

**Published:** 2021-02-26

**Authors:** Youn Jung Kim, Yejin Lee, Hong Zhang, Ji-Soo Song, Jan C.-C. Hu, James P. Simmer, Jung-Wook Kim

**Affiliations:** 1 Department of Molecular Genetics & DRI, School of Dentistry, Seoul National University, Seoul 03080, Korea; ykim71@snu.ac.kr; 2 Department of Pediatric Dentistry & DRI, School of Dentistry, Seoul National University, Seoul 03080, Korea; lyj72255621@gmail.com (Y.L.); pedosong@snu.ac.kr (J.-S.S.); 3 Department of Biologic and Materials Sciences, School of Dentistry, University of Michigan, Ann Arbor, MI 48108, USA; zhanghon@umich.edu (H.Z.); janhu@umich.edu (J.C.-C.H.); jsimmer@umich.edu (J.P.S.)

**Keywords:** whole exome sequencing, *SP6*, amelogenesis imperfecta, hereditary enamel defects, de novo mutation

## Abstract

Amelogenesis imperfecta (AI) is a heterogeneous group of rare genetic disorders affecting tooth enamel formation. Here we report an identification of a novel de novo missense mutation [c.817_818delinsAT, p.(Ala273Met)] in the *SP6* gene, causing non-syndromic autosomal dominant AI. This is the second paper on amelogenesis imperfecta caused by *SP6* mutation. Interestingly the identified mutation in this study is a 2-bp variant at the same nucleotide positions as the first report, but with AT instead of AA insertion. Clinical phenotype was much more severe compared to the previous report, and western blot showed an extremely decreased level of mutant protein compared to the wild-type, even though the mRNA level was similar.

## 1. Introduction

Amelogenesis imperfecta (AI) is a collection of hereditary enamel defects that are genetically heterogeneous and phenotypically diverse [1]. The prevalence varies from 1:700 to 1:14,000, according to the location and study population [2]. Furthermore, AI can occur as an isolated condition or as part of a syndromic phenotype [3]. Clinically, AI can be categorized as a hypoplastic, hypocalcification or hypomaturation form, depending on the quantity and quality of the affected enamel. However, often the clinical phenotype is not clear enough to derive the correct diagnosis of AI, primarily because of the inadequate number of samples for analyses or because the destruction of the tooth structure, due to attrition, abrasion, or dental caries, has obscured the clinical presentation of the enamel defects [4,5]. Therefore, the term “hypomineralization” was proposed to indicate hypocalcification and hypomaturation, together [6]. 

Mutations in genetic factors such as *DLX3* or *AMELX* expressed during early amelogenesis can have a profound impact on the entire enamel forming process, resulting in AI with both hypoplastic and hypomaturation features [7,8,9]. Furthermore, the clinical phenotype of AI may be altered by differences in genetic background and influences from environmental factors such as systemic and/or nutritional disturbances [10].

To date, more than 20 genes have been identified to cause AI [11]. Genes encoding the structural proteins (*AMELX*, *ENAM* and *AMBN*) and proteolytic enzymes (*MMP20* and *KLK4*) in the developing enamel matrix have been screened initially, and mutations have been identified [12,13,14,15,16]. With the remarkable advances in modern genetic techniques, such as whole exome or genome sequencing, many genes with little-known or unknown functions in amelogenesis have been successfully identified [4,17].

Recently, the involvement of a novel gene, *SP6* (aka, Epiprofin or *EPFN*), was identified in a Caucasian family to cause autosomal dominant hypoplastic AI [18]. In this study, we report the identification of the second *SP6* mutation in a Korean AI family and present a unique clinical phenotype.

## 2. Materials and Methods

### 2.1. Study Subjects and Informed Consent

The institutional review board of the Seoul National University Dental Hospital reviewed and approved the study protocol (CRI05003G), and informed consent was obtained from each individual or a guardian. Clinical and radiographic examination were performed

### 2.2. Clinical Examination and Sample Collection

The proband, mother and father were examined for complete oral and systemic evaluation. The older brother of the proband was reportedly healthy, without any tooth symptoms, and could not attend the examination; therefore, a saliva DNA collection kit (Oragene OG-250, DNAgenotek, Ottawa, Ontario, Canada) was given to him. Peripheral whole blood was collected from the other family members. 

### 2.3. DNA Isolation and Whole Exome Sequencing

Genomic DNA was isolated from 2 ml of peripheral whole blood or saliva using the NucleoSpin genomic DNA purification kit (Macherey-Nagel GmbH & Co., Düren, Germany). The purified DNA was measured for quality and quantity by spectrophotometry measured by the OD_260_/OD_280_ ratio. To further check the integrity of the isolated DNA, agarose gel electrophoresis was performed. Whole exome sequencing was performed with the Agilent SureSelect XT Human All Exon V5 Target Enrichment System, and paired-end sequencing reads were obtained with the Illumina HiSeq 2500 (Theragen Etex Bio Institute, Suwon-si, Korea).

### 2.4. Bioinformatics 

A series of bioinformatic analysis programs were applied to the obtained paired-end sequencing reads. The sequencing reads were trimmed to remove the adapter sequences using Cutadapt and aligned to the reference human genome assembly hg38 with the Burrows-Wheeler Aligner. Samtools, Genome Analysis Tool Kit, and Annovar were used to obtain a list of sequence variants. Sequence variants were annotated with dbSNP build 147.

### 2.5. Sanger Sequencing 

The identified mutation in the proband was confirmed by Sanger sequencing with the following primers (676 bp, sense: 5′-GGGCTAAGGCCTTGGAAGTA-3′, antisense: 5′-AAATACGCACCTTCCCCTCT-3′). Sanger sequencing was performed for all four participating family members.

### 2.6. Cloning of the Wild-Type and Mutant SP6 Expression Vectors

The wild-type *SP6* coding sequence with flag tagging was cloned from the genomic DNA with the following primers (sense: 5′-AAGCTTGATCCCGGCAATGCTAACCGC-3′, antisense: 5′-CAGCGTGGCTCCCTCCAACTCAGCCGACTACAAAGACGATGACGACAAGCTCGAG-3′), and the mutation was introduced by conventional PCR mutagenesis (sense: 5′-ACGTCGCACCTGAAGATGCACCTGCGCTGG-3′, antisense: 5′-CCAGCGCAGGTGCATCTTCAGGTGCGACGT-3′). Wild-type and mutant sequences were subcloned into the pcDNA3.1 vector with HindIII and XhoI restriction endonucleases.

### 2.7. Western Blot and RT-PCR of the Wild-Type and Mutant SP6

COS-7 cells in 35 mm culture dishes were transfected with the wild-type and mutant *SP6* vectors. After 30 hours post-transfection, the cells were harvested with RIPA buffer with protease inhibitors. Primary antibodies for flag (Sigma-Aldrich, MO, USA) and GAPDH (abm, Richmond, BC, Canada) were used at a titer of 1:10,000 and incubated at 4 °C overnight. The secondary antibody of goat anti-mouse (Thermo, Waltham, MA, USA), conjugated with HRP, was used at a titer of 1:10000. Total RNA was isolated with the RNeasy mini Kit (Qiagen, Germantown, MD, USA), and cDNA was synthesized. RT-PCR was performed using the *SP6* primers (530 bp, sense: 5′-GGTAACCTGCGAGGACCTG-3′, antisense: 5′-GCTTCTTCTTGCCCCCATC-3′) and the *GAPDH* primers (309 bp, sense: 5′-CCAAGGTCATCCATGACAAC-3′, antisense: 5′-GCTTCACTACCTTCTTGATG-3′).

## 3. Results

**Figure 1:**
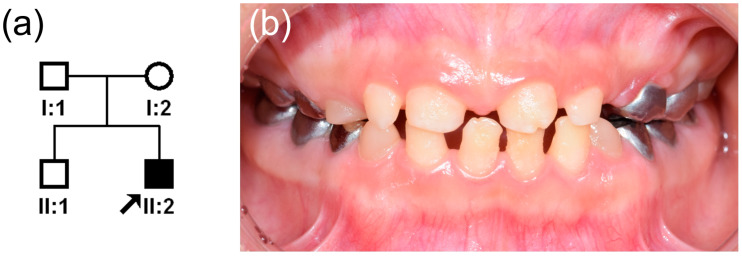
(**a**) Pedigree of the study family. The black arrow denotes the proband. (**b**) Clinical photo of the central occlusion of the proband taken at age 9 years 9 months. Hypoplastic enamel resulted in small and misshapen anterior permanent teeth with wide interdental spacing.

**Figure 2:**
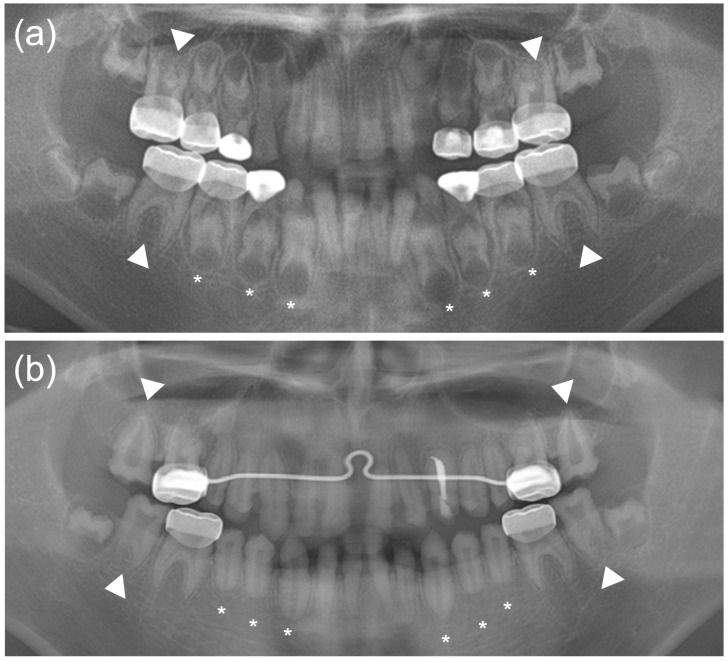
(**a**) A panoramic radiograph of the proband taken at age 9 years 9 months. The first permanent and deciduous molars have been treated by stainless steel crown restoration. Severe hypoplastic enamel can be seen in all teeth. An unusual rocket-shaped form of the developing canines and premolars can be observed (asterisks). The first permanent molars exhibit taurodontism (white arrow heads). (**b**) A panoramic radiograph of the proband taken at age 13 years 9 months. Erupted canines and premolars show a blunt root apex with enlarged pulp chambers (asterisks). The taurodontism is also clearly seen in the second molars (white arrow heads).

The proband was a nine-year-old boy with hypoplastic AI who was referred from a local dental clinic. He was the second child from a non-consanguineous marriage and uneventful pregnancy and delivery (Figure 1a). His height and weight were slightly small compared to the same age group. His height (130.2 cm) was 9.5 percentile and his weight (26.5 kg) was 7.3 percentile, according to the Korean reference growth chart. He received surgery to correct bilateral blepharoptosis when he was six years old. Otherwise, his past medical history was unremarkable, and there was no other individual having similar dental features on the paternal or maternal sides of the family. To manage the rapidly wearing tooth structure, the first permanent molars and deciduous molars were treated with stainless steel crowns. The hypoplastic enamel, resulting in small teeth with wide interdental spacing, can be observed in the anterior region of the mouth (Figure 1b). Panoramic radiograph examination revealed severe hypoplastic enamel in all developing teeth and uniquely peculiar rocket-shaped developing roots of the canines and premolars (Figure 2a). After full eruption, these teeth showed a blunt root apex with enlarged pulp chambers. The first permanent molars exhibited taurodontism and a vertically extended pulp chamber resulting in short roots. Later, the taurodontism was also clearly seen in the second molars (Figure 2b). 

**Figure 3:**
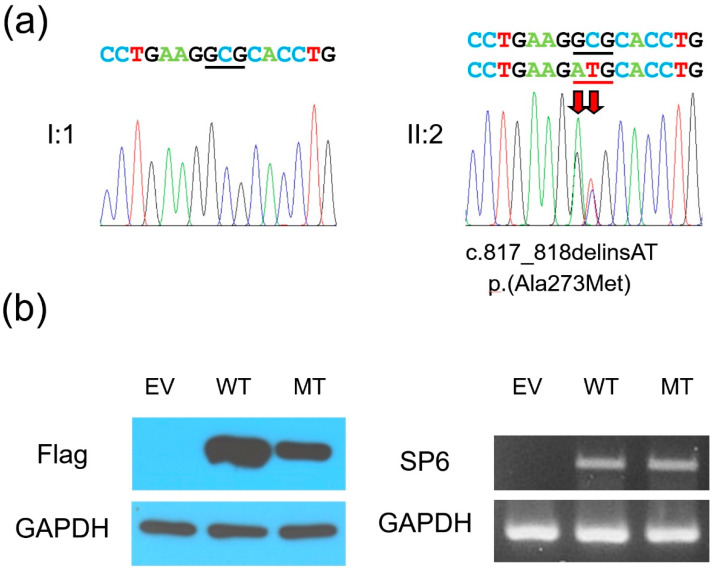
(**a**) DNA sequencing chromatograms of the PCR amplification products from the father (I:1) and the proband (II:2). The wild-type (top) and the mutated nucleotide sequences (bottom) are shown above the chromatograms. Red arrows indicate the location of the mutation [c.817_818delinsAT, p.(Ala273Met)]. Nucleotides encoding the amino acid at codon position 273 are underlined with black (wild-type allele) and red (mutant allele) lines. (**b**) Western blot and RT-PCR results. The C-terminal flag-tagged wild-type and the mutant human *SP6* genes in the pcDNA3.1 vectors were transfected into COS-7 cells. After 30 hours, the cells were harvested for protein and total RNA collections. Western blot using flag antibody showed weak expression of mutant SP6 protein (left images), but the mRNA expression level was similar between the wild-type and mutant form (right images). EV: empty vector, WT: wild-type, MT: mutant.

Whole exome sequencing and trio analysis identified a likely pathogenic variant (a de novo *SP6* mutation) without any of the other mutations in the known AI-causing genes (Table 1). The identified mutation was a 2-bp insertion and deletion mutation (NM_199262.3: c.817_818delinsAT), which would be predicted to change the highly conserved amino acid alanine to methionine at codon position 273 [NP_954871.1: p.(Ala273Met)] (Figure 3a). This mutation was not listed in the Exome Aggregation Consortium (ExAC) and gnomAD databases. In silico prediction programs, PolyPhen-2 and SIFT predicted probably damaging with a score of one and deleterious with a score of zero, respectively. Furthermore, the protein expression study with western blot revealed that the expression of the mutant protein was reduced compared to the wild type, even though their mRNA levels were similar (Figure 3b).

## 4. Discussion

The role of the Sp6 transcription factor, *Sp6*, in tooth morphogenesis has been demonstrated in the mouse *Sp6* knockout model [19,20]. *Epfn*^-/-^ mice exhibited supernumerary teeth, deficient enamel, defective cusp and root formation, delayed eruption, and abnormal dentin structure, along with defects in several epithelium-containing organs or appendages, such as skin, hair follicles, and digit formation. Identification of a 2-bp insertional *Sp6* mutation in the spontaneous amelogenesis imperfecta rat model with autosomal recessive inheritance [21] further suggested *SP6* as a candidate gene for human AI with or without other non-oral defects.

Recently, a missense *SP6* mutation was identified in a Caucasian family to cause autosomal dominant hypoplastic AI [18]. The mutation was a 2-bp variant (c.817_818GC>AA) that changed the highly conserved alanine to lysine [p.(Ala273Lys)] in the first of the three zinc finger domains. The mutation was predicted to alter the DNA-binding ability, and the mutant protein was demonstrated to have a reduced binding ability and faster dissociation rate compared to the wild-type when interacting with the *AMBN* promoter sequence. The clinical phenotype was characterized by generalized hypoplastic AI with an irregular surface on all teeth, and there were no other oral and non-oral defects. 

In this study, whole exome sequencing and trio analysis identified a de novo *SP6* mutation. Interestingly, the identified mutation was a 2-bp variant at the same nucleotide positions as the previous report [18] but with an AT instead of an AA insertion (c.817_818delinsAT), changing alanine to methionine [p.(Ala273Met)]. 

There are some differences in phenotype between the proband of this study and the affected individuals from the previous report, even though the same codon was mutated. The proband in this study with the de novo mutation has an unusual root development, taurodontism, and severe hypoplastic AI, while the generalized hypoplastic AI with an irregular enamel surface, representing a mild phenotype, was evident in the previous report. Recently, the *Msx2-Sp6-Follistatin* pathway, controlling the ameloblast life cycle and amelogenesis, has been demonstrated in a mouse model [22]. In addition, the phenotype of the murine knockout models suggests the involvement of the Sp6 transcription factor in dentin formation. In addition to the reduced DNA binding ability demonstrated in the previous study, the apparently low level of mutant protein expression in this case is likely responsible for the severe dental phenotypes. However, it is not clear that the *SP6* mutation examined in this study is solely responsible for the unique root development and taurodontism because there can be other contributing factors. Therefore, further studies are necessary to find the functional role of SP6.

X-linked hypoplastic AI can be caused by mutations in *AMELX* [23]. Autosomal dominant hypoplastic AI can be caused by mutations in *ENAM*, *COL17A1,* and *LAMB3* [3,24]. While mutations in *AMBN* and *ACP4* cause non-syndromic autosomal recessive hypoplastic AI [14,17], mutations in *FAM20A* cause syndromic autosomal recessive hypoplastic AI (enamel-renal syndrome, OMIM#204690) [25]. This study confirmed that the *SP6* mutation causes hypoplastic AI in an autosomal-dominant inheritance patterns, in contrast to the mouse phenotype. 

In conclusion, we identified a novel de novo mutation in the *SP6* gene in a hypoplastic AI family with a peculiar developing root form and taurodontism. This study will expand the mutational spectrum of rare *SP6* mutations and expand our understanding of the involvement of SP6 in tooth development.

## Figures and Tables

**Table 1:** Statistics for whole exome sequencing.

Sample	Total Reads	Mapping Rate (%)	Median Target Coverage	Coverage of Target Region (%)	Fraction of Target Covered with at Least	
					20X	10X
I:1	69,365,294	99.6	95	96.5	93.5	95.5
I:2	68,921,817	99.6	87	96.3	93.0	95.3
II:2	59,890,807	99.7	82	96.5	92.8	95.4

## Data Availability

The data presented in this study are openly available in ClinVar (http://www.ncbi.nlm.nih.gov/clinvar), Submission ID: SUB9143361.
